# Extending statistical models for source attribution of zoonotic diseases: a study of campylobacteriosis

**DOI:** 10.1098/rsif.2018.0534

**Published:** 2019-01-30

**Authors:** Sih-Jing Liao, Jonathan Marshall, Martin L. Hazelton, Nigel P. French

**Affiliations:** 1 School of Fundamental Sciences, Massey University, Palmerston North 4442, New Zealand; 2 mEpiLab, Infectious Disease Research Centre, School of Veterinary Science, Massey University, Palmerston North 4442, New Zealand; 3 New Zealand Food Safety Science & Research Centre, Massey University, Palmerston North 4442, New Zealand

**Keywords:** source attribution, *Campylobacter*, genetic model, Dirichlet, DIC

## Abstract

Preventing and controlling zoonoses through the design and implementation of public health policies requires a thorough understanding of transmission pathways. Modelling jointly the epidemiological data and genetic information of microbial isolates derived from cases provides a methodology for tracing back the source of infection. In this paper, the attribution probability for human cases of campylobacteriosis for each source, conditional on the extent to which each case resides in a rural compared to urban environment, is estimated. A model that incorporates genetic data and evolutionary processes is applied alongside a newly developed genetic-free model. We show that inference from each model is comparable except for rare microbial genotypes. Further, the effect of ‘rurality’ may be modelled linearly on the logit scale, with increasing rurality leading to the increasing likelihood of ruminant-sourced campylobacteriosis.

## 1. Introduction

Modelling of disease surveillance data to explore patterns of infectious diseases has had a long history in public health. Infectious diseases can cause high economic and medical costs due to morbidity and mortality. In recent decades, the annual number of global deaths caused by infections has levelled off at approximate 15 million and may remain at this level for the next three decades [1,2]. In order for such an enormous health burden to be reduced, preventing and controlling infectious diseases becomes extraordinarily important, and our ability to intervene depends on how much we know about the nature of disease transmission.

For zoonotic diseases, transmission to humans from animal reservoirs may be complex, involving many sources and exposures linked by different pathways, via food, water, through environmental contamination or direct contact with animals. Knowledge of the potential sources and pathways of infection is key to reducing the burden of disease. For instance, infected wild birds may contaminate environmental water and cause disease spread to water users, either humans or other animals [3]. Tracing the source of infection becomes crucial to increasing the ability to implement risk management and intervention [4,5].

Modelling zoonoses requires an advanced approach with the focus changed from just epidemiology to a combination of epidemiology, evolutionary genetics and biology [6]. Some source attribution models have been proposed to estimate the number of cases attributable to different sources by using epidemiological information and the association with genotypes found in humans and sources [7–10]. The genetic information used in such integrated models is typically derived from molecular genotyping that groups closely related organisms together [11]. A common method used is multilocus sequence typing (MLST) [12–14], which uses nucleotide sequences of internal fragments of a small set of housekeeping genes. Such sequences have sufficient variation to distinguish differing pathogen lineages, while being relatively stable within lineages. Each unique nucleotide sequence (allele) at each housekeeping gene (locus) is assigned a number, and the set of numbers across all loci (the allelic profile) is then taken as the genotype, which is assigned a sequence type (ST) number.

For the pathogen *Campylobacter* which causes campylobacteriosis, a worldwide gastrointestinal disease in humans, the commonly used seven-gene MLST scheme consists of housekeeping genes *asp*A (aspartase A), *gln*A (glutamine synthetase), *glt*A (citrate synthase), *gly*A (serine hydroxymethyltransferase), *pgm* (phosphoglucomutase), *tkt* (transketolase) and *unc*A (ATP synthase *α* subunit). An illustrative example of MLST data for *Campylobacter* is presented in table 1. It shows that the genotypes ST-2026 and ST-474 have different allelic combinations across all seven loci, while ST-403 differs from ST-2026 only at *gln*A, and ST-2343 differs from ST-474 at *glt*A and *pgm*. The different allelic profiles enable comparison of gene similarities or dissimilarities so that an association between sources and infected cases can be made, by comparing the distribution of genotypes from human cases with those from potential reservoirs.

**Table 1.** The allelic profiles of a selection of genotypes, composed of seven allele numbers at each of the seven housekeeping genes.

genotype	*asp*A	*gln*A	*glt*A	*gly*A	*pgm*	*tkt*	*unc*A
ST-403	10	27	16	19	10	5	7
ST-474	2	4	1	2	2	1	5
ST-2026	10	1	16	19	10	5	7
ST-2343	2	4	5	2	10	1	5

Human campylobacteriosis is caused mainly by *C. jejuni* and *C. coli* which are the dominant species associated with approximately 80% and 15% of illnesses respectively [15]. Common symptoms of infection are diarrhoea, abdominal pain and fever; however, a severe complication named Guillain–Barré syndrome may develop, which is a life-threatening disease that weakens the nervous system and leads to paralysis of the limbs and respiratory failure [16]. The pathogen can be spread between animals, or from animals and wild birds to humans. Transmission routes may be via drinking contaminated water, eating undercooked animal food products, or handling animal food products that are already contaminated by faeces.

The first step for attribution models that use genetic information is building the sampling distribution of genotypes among each putative source. This may range from using the proportion of each observed genotype [9] on each source through to using allelic profile information to derive mutation and recombination rates within each source, and migration rates between each source [4]. A key question is whether more complex genetic models yield superior attribution results or whether a significantly simpler model may suffice, but few authors in the literature have addressed this point. This becomes more important as model complexity extends to include epidemiological covariates. We are therefore motivated to develop a simple model in order to assess the additional information that the more complex models provide by using data originating from a study on human campylobacteriosis conducted in New Zealand [17].

In this study, we develop statistical models for source attribution and demonstrate their use on the campylobacteriosis study. We compare the performance of the asymmetric Island model [4], which considers genetic evolution when estimating the genotype sampling distribution on each source, to a simple model that uses only the prevalence of each type to derive the sampling distribution. This comparison brings into sharp focus the contribution of the asymmetric Island model to the overall analysis, enhancing our understanding of the operation of these models and facilitating model checking. We then extend both models in a Bayesian context to incorporate covariates, exploring the effect of human case rurality on attribution results via a linear trend on the logit scale or with separate categories, and performing model comparison.

## 2. Material and methods

### 2.1. MLST data

Our data originate from the campylobacteriosis study and comprise microbial genotype information from each observed human case obtained from analysis of stool samples and also from a pool of non-human cases corresponding to potential zoonotic sources of disease. These samples were obtained at a surveillance sentinel in the Manawatu region of New Zealand from March 2005 to December 2014. Further details can be found in [17]. Briefly, the data contain 1460 isolates taken from human cases, and 2128 isolates sampled from chicken carcases, cattle, sheep, environmental water, wild birds and so on, over the same time period and from the same geographical location. The non-human samples were categorized into four groups representing major sources of infection: poultry, ruminants, water and others (consisting of cats, dogs and various wild birds). The total number of unique genotypes from all isolates is 348, with 36% of genotypes found among human cases. Table 2 lists five common genotypes found in human and source isolates, the first four of which are frequently observed in human cases. As found in other studies, ST-45 and ST-474 are detected mainly in poultry, while ST-42 and ST-2026 are detected mainly in ruminants [6,10,13]. The fifth genotype, ST-2381 is not found among human cases, appearing only in the water and other sources, in this case being found in Pukeko and Takahē birds from the Rallidae family [18,19].

**Table 2.** The frequency of five genotypes found from human and four source isolates.

genotype	human	poultry	ruminants	water	others
ST-42	59	7	53	10	2
ST-45	149	155	10	21	54
ST-474	247	60	15	5	9
ST-2026	28	0	40	5	2
ST-2381	0	0	0	60	3

### 2.2. Location information of human cases

**Figure 1.**
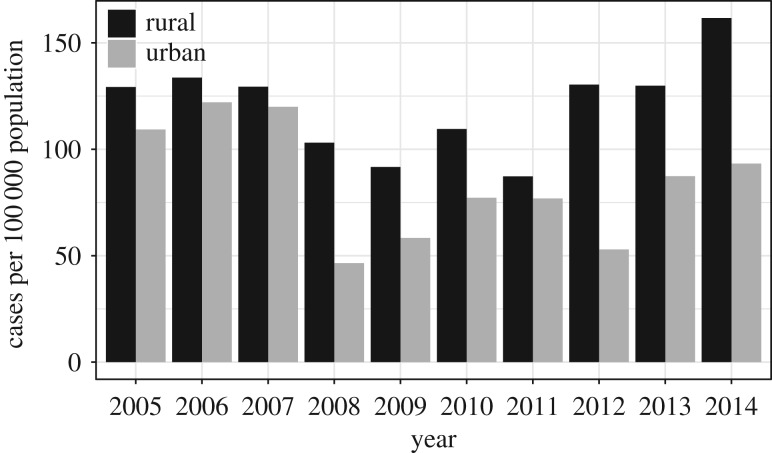
Case rates per 100 000 population in urban and rural areas of the Manawatu region of New Zealand from 2005 through 2014. An intervention in the poultry industry conducted in 2007 and 2008 resulted in a decreasing incidence of campylobacteriosis in the following years, particularly in urban areas.

The data also contain location information, in the form of an ordinal classification of urban and rural areas with seven levels coded from − 3 to 3: highly rural/remote area, rural area with low urban influence, rural area with moderate urban influence, rural area with high urban influence, independent urban area, satellite urban area and main urban area. Approximately 8% of individuals in the Manawatu dataset have no information about the location, which we assume are missing at random. Table 3 lists the remainder of typed human cases in each classification of rurality as well as the population from the 2006 and 2013 Census [20,21]. The case rate per 100 000 population is illustrated in figure 1, where we see the burden of infection in urban areas drop remarkably from 2008, coinciding with an intervention in the poultry industry implemented by the New Zealand Food Safety Authority (NZFSA) in 2007 and 2008. It shows the intervention improved infection rates in urban areas; however, it only has a temporary effect in rural areas.

**Table 3.** The number of human cases in each rurality class during 2005–2014, and the population size in 2006 and 2013, in the Manawatu region of New Zealand.

rurality scale	description	human cases	2006	2013
−3	highly rural/remote area	16	1572	1527
−2	rural area with low urban influence	103	8382	8316
−1	rural area with moderate urban influence	124	10 392	10 734
0	rural area with high urban influence	78	6579	7155
1	independent urban area	240	28 611	28 188
2	satellite urban area	187	19 725	20 526
3	main urban area	596	76 047	78 108

### 2.3. Genotype models with and without microbial genetic information

The goal of attribution models is to estimate the probability that the observed human cases arise from each putative source. Given the genotyping information, we first estimate the sampling distribution of genotypes for each source and then estimate the appropriate combinations of those genotype distributions that most likely give rise to the set of genotypes observed among human cases. Specifying first the sampling distribution of genotypes found on sources is fundamental for the purpose of not only exploring how it affects the source attribution probability but also investigating the difference in attribution effect made between different genetic models.

Suppose we have isolates collected from human and non-human cases, of which *H* isolates belong to humans, and the remaining *N* isolates are categorized in *J* groups as the major sources attributed to the infection. Let *I* genotypes be the total number of unique types detected from all isolates and denote *n*_*j*_ as the marginal frequency of types found in source *j*, where $\sum_{j}^{J}n_{j}=N$. Typically, the number of detected types *I* is smaller than the sample size of isolates as multiple isolates will be of the same type.

Each type *i*, *i* = 1, …, *I*, may be found in more than one human case and so we model the likelihood of observing human cases with genotype ST_*i*[*h*]_ using a multinomial distribution, in which *i*[*h*] is the index of the ST found in human case *h*. The likelihood via the law of total probability may be expressed as

2.1 $$\begin{array}{rl} & L({\text{ST}}_{i[1]},{\text{ST}}_{i[2]},\ldots ,{\text{ST}}_{i[H]})=\prod_{h=1}^{H}\sum_{\;j=1}^{J}p({\text{ST}}_{i[h]}|\text{source }j)p(\text{source}\;j), \end{array}$$

where $p({\text{ST}}_{i[h]}|\text{source}\;j)$ is the probability that genotype ST_*i*_ found in human case *h* arises from the sampling distribution of source *j*, and *p*(source *j*) is the attribution probability that a random human case is infected from source *j*. Given we know $p({\text{ST}}_{i[h]}|\text{source}\;j)$, estimation of *p*(source *j*) may be found by optimizing the likelihood (2.1), for example, using a Metropolis–Hastings algorithm within a Bayesian context, with suitable priors on *p*(source *j*).

The asymmetric Island model [4] adopted in the source attribution study for human campylobacteriosis [17] uses the allelic profile information for each genotype in an evolutionary model, estimating mutation and recombination probabilities within, and migration probabilities between, each source ‘island’. It thus estimates $p({\text{ST}}_{i[h]}|\text{source}\;j)$ indirectly, by first estimating the evolutionary parameters, and then deriving the sampling distributions. This allows the asymmetric Island model to estimate the likelihood of observing a genotype on a source when it has not been previously observed.

To discover the effect of incorporating genetic information at the allelic profile level as used in the asymmetric Island model, a simple model is developed for the genotype sampling distribution. With the assumption that the observed distribution of genotypes is representative of the true distribution, we model observed genotypes using a multinomial distribution. Let *x*_*ij*_ denote the count of genotype ST_*i*_ found in source *j* with probability *π*_*ij*_, where *i* = 1, …, *I* and *j* = 1, …, *J*. To make inference about *π*_*ij*_, the likelihood is of a multinomial form,

$$L(\pi_{\;j};x_{\;j})=\frac{n_{j}!}{\prod_{i=1}^{I}x_{ij}!}\prod_{i=1}^{I}\pi_{ij}^{x_{ij}},$$

where *x*_*ij*_ can be 0, indicating genotypes are not observed on the sources; $n_{j}=\sum_{i=1}^{I}x_{ij}$ is the total count of all types found on source *j* and *π*_*ij*_ is subject to $\sum_{i=1}^{I}\pi_{ij}=1$, and 0 ≤ *π*_*ij*_ ≤ 1. As the family of Dirichlet distributions is a conjugate pair for the multinomial distribution, assume the prior for ***π***_*j*_ follows a Dirichlet density with parameters ***γ***_*j*_,

$$p(\pi_{\;j})\propto \prod_{i=1}^{I}\pi_{ij}^{\gamma_{ij}-1}.$$

Then the posterior for ***π***_*j*_ takes the form of a Dirichlet probability function with parameters (***γ***_*j*_ + ***x***_*j*_ − ***1***),

$$\begin{array}{rl} \;p(\pi_{\;j}|x_{\;j}) & \propto L(\pi_{\;j};x_{\;j})p(\pi_{\;j})\\ & \propto \prod_{i=1}^{I}\pi_{ij}^{\gamma_{ij}+x_{ij}-1},\;\gamma_{ij}>0. \end{array}$$

To express the belief that every isolate is equally likely *a priori*, the parameter of the Dirichlet prior is assumed as ***γ***_*j*_ = ***1***. Therefore, $p({\text{ST}}_{i[h]}|\text{source}\;j)$ can be obtained by simulating from the Dirichlet posterior.

### 2.4. Model fitting on rurality scale

Previously we described how to estimate the marginal probabilities that a randomly selected human case is due to a given source. To estimate the attribution probability, 100 posterior samples of $p({\text{ST}}_{i[h]}|\text{source}\;j)$ are generated using the asymmetric Island or Dirichlet models. For each posterior sample, we infer *p*(source *j*) using the likelihood (2.1). This has the effect of integrating over the uncertainty in $p({\text{ST}}_{i[h]}|\text{source}\;j)$ when estimating *p*(source *j*).

To extend this analysis so as to include individual level covariates, we need to calculate subject-specific attribution (conditional) probabilities, p(source j|covariates). To that end, let *F*_*jh*_ denote the attribution probability of source *j* for the *h*^th^ human case, with constraints $\sum_{\;j=1}^{J}F_{\;jh}=1$ and 0 ≤ *F*_*jh*_ ≤ 1, where *h* = 1, …, 1460 and *j* = 1, …, 4. We model the probabilities *F*_*jh*_ using a linear model on the logit scale, that is,

2.2 $$F_{\;jh}=\frac{\exp(f_{\;jh})}{\sum_{\;j=1}^{4}\exp(f_{\;jh})},$$

where *f*_4*h*_ = 0 is treated as the baseline of *f*_*jh*_. Consider the case where the genotype data for each human case are supplemented by *p* additional variables. A general model of *f*_*jh*_ with linear combinations of the variables, *c*_1_, …, *c*_*p*_, then has the form

$$f_{\;jh}=\alpha_{\;j}+\beta_{\;j1}c_{1h}+\beta_{\;j2}c_{2h}+\cdots +\beta_{\;jp}c_{\;ph},$$

for the *h*th individual. Note that if there is a single categorical variable with *L* levels, then *F*_*jh*_ and *f*_*jh*_ will take no more than *L* distinct values. In a slight abuse of notation, we will at times refer to *F*_*jh*_ in which the *h* index refers to the factor level, rather than to a particular subject at that level.

To apply the general model of *f*_*jh*_ to the campylobacteriosis data, assume *z* is the variable ranging from − 3 to 3 representing the classified rurality of each human case. Then two ways of treating the variable *z* in model fitting are proposed: one is to treat it as numeric, and the other as categorical. To differentiate the performance between the two fitted models, we link ‘the linear model’ and ‘the categorical model’ to the first and the latter fitted model, respectively. Hence, the linear prediction function for source *j* for each human case given the degree of rurality is a numeric variable and can be written as

2.3 $$f_{\;jh}=\alpha_{\;j}+\beta_{\;j}z_{h},$$

where *z*_*h*_ can be any number of the seven scales if case *h* was from such a degree of rurality. Conversely, if we treat each of the seven rurality degrees as an indicator with a superscript number *d*, which corresponds with the position of the category ranged from − 3 to 3, the model (2.3) can be rewritten as

2.4 $$f_{\;jh}=\beta_{1j}z_{1h}+\beta_{2j}z_{2h}+\cdots +\beta_{dj}z_{dh}+\cdots +\beta_{7j}z_{7h},$$

where

$$z_{dh}=\{\begin{array}{rl} 1 & \;\text{if case }\;h\;\text{is in the category }\;d,\\ 0 & \;\text{otherwise}. \end{array}$$

As a consequence, the estimated attribution probabilities are obtained via equation (2.2) after fitting the data to model (2.3) or to model (2.4).

### 2.5. Markov chain Monte Carlo algorithm

In the interest of quantifying the uncertainty of the posterior attribution probability, we perform Bayesian inference for source attribution probabilities using Markov chain Monte Carlo (MCMC) methods. Assume the priors on parameters of interest in model (2.3) and model (2.4) follow a standard normal distribution. Let ***θ*** denote the vector of parameters, with elements *θ*_*t*_ for *t* = 1, …, *T*. For example, ***θ*** in model (2.3) and model (2.4) can be {*α*_1_, *α*_2_, *α*_3_, *β*_1_, *β*_2_, *β*_3_} and {*β*_11_, *β*_12_, …, *β*_*dj*_, …, *β*_73_}, respectively. To update *θ*_(*t*)_ and hence *f*_*jh*_ and *F*_*jh*_, we use the Metropolis–Hastings algorithm in a Markov chain with a length of 11 000 iterations. The first 1000 samples are removed as the burn-in period (during which time the chain converges) and the sequence is thinned every 100th sample to reduce computer storage. Here are the steps in detail:

(0) Sample *T* random values from *N*(0, 1) as initial values of the parameter set ***θ*** for model (2.3) or model (2.4).
(1) Sample a permutation *P*_*T*_ of {1, …, *T*}.
(2) For each *t* ∈ *P*_*T*_:
  (a) Propose a candidate $\theta^{*}$ with *θ*_(*t*)_ updated by a normal proposal distribution, $Q(\theta_{\left(t\right)}^{*},\theta_{\left(t\right)})=N(\theta_{\left(t\right)},1)$.
  (b) Use $\theta^{*}$ to calculate a new set of $f^{*}$ for source *j*, *j* = 1, 2, 3, via model (2.3) or model (2.4) and find the associated $F^{*}$ for each case by putting the vector $\left(f^{*},f_{4}=0\right)$ in equation (2.2).
  (c) Compute the acceptance probability *a* = min{1, *g*}, where
    $$g=\frac{L(F^{*};\text{ST})}{L(F;\text{ST})}\frac{Q(\theta_{\left(t\right)}|\theta_{\left(t\right)}^{*})}{Q(\theta_{\left(t\right)}^{*}|\theta_{\left(t\right)})}\frac{\;p(\theta_{\left(t\right)}^{*})}{p(\theta_{\left(t\right)})},$$
    in which the likelihood $L(F^{*};\text{ST})$ is given by equation (2.1).
  (d) Accept the proposals $f^{*},F^{*}$ with probability *a*.
(3) Repeat from step 1 for the given number of iterations.

## 3. Results

### 3.1. Posterior attribution probability

**Figure 2.**
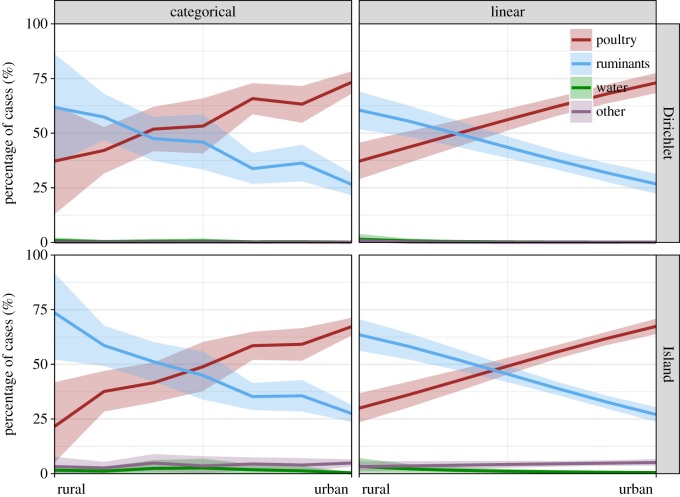
Posterior mean attribution (*F*) of human cases with 80% credible intervals for source: poultry, ruminants, water and others over the rurality scales from highly rural areas to main urban areas (table 3). The attribution is generated from both the linear and the categorical models, given the sampling distribution of genotypes with evolutionary information (the asymmetric Island model) or without any genetic information (the Dirichlet model). (Online version in colour.)

Posterior attribution of human cases of campylobacteriosis (*F*) with 80% credible intervals for each source is illustrated for each rurality grade in figure 2. The graphs are categorized by the types of model (asymmetric Island or Dirichlet) and the manner in which the rurality variable is modelled (categorical or linear on the logit scale).

Overall, the attribution results are relatively stable irrespective of the types of model or how rurality is represented in the attribution model. The majority of human cases are attributed to ruminants and poultry, with more cases attributed to ruminants in rural areas and more cases attributed to poultry in urban areas. For the Dirichlet and asymmetric Island models, the linear and categorical models of rurality show broadly the same trend, suggesting that the additional flexibility given by the categorical model is not required and that the shift in attribution as rurality changes is adequately modelled by a linear trend on the logit scale. The linear model has the advantage of tighter credible intervals as it can share data across the seven levels of rurality, resulting in a clearer separation of ruminant and poultry attribution, particularly in highly rural areas where the data are sparse. There are some small differences between the genotype models, with the Dirichlet model showing a greater attribution to poultry (ranging from 40% in highly rural areas to 75% in main urban centres) than the asymmetric Island model (ranging from 30% in rural areas to 65% in urban centres). This also occurs similarly in the categorical model.

Interestingly, the asymmetric Island model attributes approximately 7% of human cases across all rurality levels to sources other than poultry, ruminants and water and gives a small attribution to water in highly rural areas, while the Dirichlet model indicates that both these sources are unimportant.

### 3.2. Model selection

We use deviance information criterion (DIC) for model comparison. DIC values obtained from our MCMC runs are displayed in table 4. Overall, there is a clear signal that a linear representation of rurality (on the logit scale) is adequate due to relatively small values compared to the categorical model. Note that the asymmetric Island and Dirichlet models are not directly comparable by DIC as the likelihoods are on different scales: the Dirichlet model assumes all potential sequence types have been observed so that $\sum_{j}p(\pi_{j})=1$, whereas the asymmetric Island model allows for unobserved sequence types so that $\sum_{j}p(\pi_{j})<1$.

**Table 4.** DIC values for the linear model and for the categorical model applied to the data from 2005 to 2014 given the sampling distribution of genotypes derived from the asymmetric Island model or Dirichlet model.

fitted models	genotype models	
	Dirichlet	Island
linear	10 968.3	12 276.4
categorical	10 976.4	12 287.2

### 3.3. Investigation between genotype models

**Figure 3.**
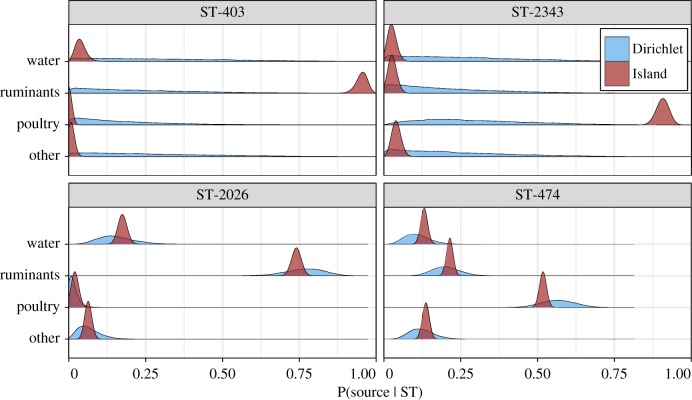
Posterior probability for each source for four sequence types from the asymmetric Island and Dirichlet models, assuming that each source is *a priori* equally likely. (Online version in colour.)

The small differences in attribution observed between the asymmetric Island and Dirichlet models may be due to the additional genetic information available to the first model. To illustrate this, figure 3 shows the probability of source given a selection of four genotypes, assuming *a priori* that each source was equally likely. Genotypes ST-403 and ST-2343 are observed primarily in humans (six cases each), with ST-403 not being observed among the sources, and ST-2343 being observed once in poultry so that the Dirichlet model has little information available to distinguish between sources. The asymmetric Island model, however, can exploit the genetic relationship between genotypes. ST-403 differs at just one locus from ST-2026, a type observed frequently in human cases and ruminant isolates, while ST-2343 differs at two loci from common genotype ST-474, observed frequently in human cases and poultry isolates (tables 1 and 2). Thus, the asymmetric Island model can clearly assign ST-403 to ruminants and ST-2343 to poultry, while the Dirichlet model cannot distinguish between sources. By contrast, both models provide similar probabilities for ST-2026 and ST-474 which are both observed frequently.

### 3.4. Robustness analysis

**Figure 4.**
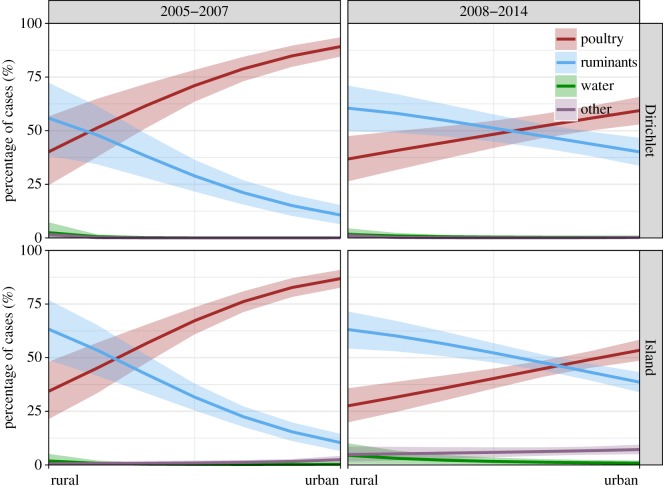
Posterior mean attribution (*F*) of human cases during 2005–2007 and 2008–2014 with 80% credible intervals for poultry, ruminants, water and other sources over the rurality scales from highly rural areas to main urban areas (table 3). The attribution is generated using the linear model given the sampling distribution of genotypes with evolutionary information (the asymmetric Island model) or without any genetic information (the Dirichlet model). (Online version in colour.)

As noted previously, a major public health initiative in 2007 led to a significant reduction in the number of cases of campylobacteriosis in New Zealand. In order to examine the effects of this change on attribution probabilities, we repeated the analysis by including an interaction, with time period 2005–2007 and 2008–2014. Figure 4 shows that the general trend in attribution by rurality for each of the time periods using a linear trend on the logit scale to incorporate rurality. There is a clear difference, with a significantly lower attribution to poultry (and correspondingly higher attribution to ruminants) in all but the most rural of areas, being strongest in highly urban areas. Thus, although the intervention did not eliminate infection arising from poultry [22], the reduction highlights the significant improvement in contribution of poultry to disease, particularly in urban areas where most cases occurred.

### 3.5. Sensitivity analysis

As with any Bayesian analysis, it is of interest to examine the sensitivity of the results to the choice of prior distributions. We originally used standard normal priors for regression coefficients on the logit scale. We also considered priors with *σ*^2^ = 4, and while this meant that *f* tended to drift further from 0, the resulting attribution probabilities *F* did not change, largely as the attribution is dominated by poultry and ruminant sources, with the water source in particular being close to zero. Thus, *f*_poultry_ and *f*_ruminants_ are positive, while *f*_water_ is negative and the magnitude of these can increase without making a significant difference to their corresponding *F*’s. The prior on *f* thus tends to restrict this ill-behaviour rather than acting as a strong constraint on attribution probabilities. The prior ***γ***_*j*_ in the Dirichlet model also makes little difference if kept small, as it most strongly effects genotypes that are rare, which do not contribute significantly to the overall attribution. The prior can be thought of as data augmentation such that ***γ***_*j*_ = ***1*** is equivalent to adding a single observation of each genotype to source *j*. Thus, large values of ***γ***_*j*_ will cause the genotype distributions across sources to look more similar, and hence result in equal attribution to each source.

## 4. Discussion

Models that determine the source of human infection, particularly for zoonotic pathogens that originate in animal populations, are of considerable value to public health policymakers. However, such models may be complex, particularly when using evolutionary models. An outstanding question is whether such complexity is required, or whether a simpler model may work as effectively. Here we developed a relatively simple model to estimate the attribution probability for each source of *Campylobacter* infection. This model differs from the asymmetric Island model, in that it does not model pathogenic evolution, opting instead to infer the sampling distribution of genotypes directly from the observed count data.

Our results show that the Dirichlet and the asymmetric Island models give largely similar final attribution probabilities, with both models demonstrating a clear effect of rurality on attribution: cases in rural areas are more likely to have originated from ruminants, while those in main urban centres are more likely to be of poultry origin. As most people in the Manawatu region live in urban centres, this highlights the importance of poultry as a reservoir for campylobacteriosis, which is well established in the literature [10,17,23,24].

When we ran models allowing attribution probabilities to differ before and after the intervention in the poultry industry in 2007, we saw a clear difference, with much lower poultry (and higher ruminant) attribution, particularly in main urban centres during 2008–2014. Considering that most people in the Manawatu region live in urban areas, and that case rates in urban areas decreased from 2008 onwards, it is clear that this intervention coincided with a dramatic reduction in poultry attributed illness as reported elsewhere [25]. Given that campylobacteriosis cases in rural areas are mostly attributed to ruminant sources, and that case rates in these areas have been higher than those in urban centres since 2008, there is a clear need for public health interventions to focus on this area.

While the overall attribution was consistent between the Dirichlet and asymmetric Island models, it would be expected that the conditional probabilities for a given genotype might differ markedly. For those genotypes observed infrequently (or not at all) among the sources, the Dirichlet model has little information while the asymmetric Island model can exploit information from cases with similar (but not identical) genetic profiles. In the case of MLST data with just seven loci, the majority of human cases and source isolates come from a relatively small number of sequence types which are observed often. Thus, the Dirichlet model performs well, as it has sufficient observations to estimate the genotype distribution well where the bulk of the data lie. It is only those genotypes that are rarely observed where it performs poorly, but as they are rarely observed, they do not contribute significantly to the overall attribution. In other circumstances, such as where we have many more than seven loci, we would expect to have many more rare genotypes, so that the Dirichlet model might provide little useful information. At the extreme example of whole genome MLST (wgMLST) where each isolate would typically be unique, it would provide essentially no information at all. In such circumstances, however, the asymmetric Island model would be expected to still perform well, assuming that information could still be transferred between similar genotypes.

The Dirichlet model is less complex than the asymmetric Island model that uses the prevalence of genotypes in sources to derive the sampling distribution of genotypes. It is similar to a recently published model, *sourceR* [8], that jointly models the source and human cases, accounting for uncertainty in the sampling process. However, *sourceR* is an extension of the Hald [9] and modified Hald [26] models which model human cases using a Poisson distribution rather than a multinomial, and instead of estimating the proportion of cases attributed to each source directly, model source effects as well as genotype effects [8,27].

Future research might focus on possible extensions to these models. One direction is to adapt the models with additional covariates, which might include age, occupation and other risk factors such as contact with animals. For example, there is evidence that children in rural areas are at higher risk of campylobacteriosis through contact with farm animals [23,28]. Another direction is in expanding the role of water. In these models, we have assumed that water is a source of human campylobacteriosis infection, but water differs from the other food and environmental sources in that it is not an amplifying reservoir for *Campylobacter* [3]. By contrast, genotypes found in water might be expected to originate in the other sources present here, particularly ruminants and wild birds, but also potentially from humans as well via discharge of unprocessed human waste. Hence, water acts as a transmission pathway from sources to humans, being both an endpoint (reduced water quality from faecal contamination) and a source (human consumption of water, either recreationally or through untreated water supplies). While there is presently little evidence that water is an important source for human campylobacteriosis from the current models, the models are fitted using sporadic cases of campylobacteriosis. However, water is known as a key source of outbreaks of campylobacteriosis, such as the large outbreak in Havelock North, New Zealand in 2016 where an estimated 5500 out of 14 000 residents became ill [29]. Thus, characterizing the source of *Campylobacter* found in water has important implications for both water quality and public health.

## Data accessibility

All codes for fitting the genotype distributions, estimating attribution probabilities and producing figures, along with the dataset are available on GitHub [30].
